# CBOL Protist Working Group: Barcoding Eukaryotic Richness beyond the Animal, Plant, and Fungal Kingdoms

**DOI:** 10.1371/journal.pbio.1001419

**Published:** 2012-11-06

**Authors:** Jan Pawlowski, Stéphane Audic, Sina Adl, David Bass, Lassaâd Belbahri, Cédric Berney, Samuel S. Bowser, Ivan Cepicka, Johan Decelle, Micah Dunthorn, Anna Maria Fiore-Donno, Gillian H. Gile, Maria Holzmann, Regine Jahn, Miloslav Jirků, Patrick J. Keeling, Martin Kostka, Alexander Kudryavtsev, Enrique Lara, Julius Lukeš, David G. Mann, Edward A. D. Mitchell, Frank Nitsche, Maria Romeralo, Gary W. Saunders, Alastair G. B. Simpson, Alexey V. Smirnov, John L. Spouge, Rowena F. Stern, Thorsten Stoeck, Jonas Zimmermann, David Schindel, Colomban de Vargas

**Affiliations:** 1 Department of Genetics and Evolution, University of Geneva, Geneva, Switzerland; 2 Centre National de la Recherche Scientifique, Unité Mixte de Recherche 7144 and Université Pierre et Marie Curie, Paris 6, Station Biologique de Roscoff, France; 3 Department of Soil Science, University of Saskatchewan, Saskatoon, Saskatchewan, Canada; 4 Department of Life Sciences, Natural History Museum, London, United Kingdom; 5 Laboratory of Soil Biology, University of Neuchâtel, Neuchâtel, Switzerland; 6 Wadsworth Center, New York State Department of Health, Albany, New York, United States of America; 7 Department of Zoology, Charles University in Prague, Prague, Czech Republic; 8 Department of Ecology, University of Kaiserslautern, Kaiserslautern, Germany; 9 Institute of Botany and Landscape Ecology, University of Greifswald, Greifswald, Germany; 10 Department of Biochemistry and Molecular Biology, Dalhousie University, Halifax, Nova Scotia, Canada; 11 Botanischer Garten und Botanischer Museum Berlin-Dahlem, Freie Universität Berlin, Berlin, Germany; 12 Institute of Parasitology, Biology Centre, Czech Academy of Sciences, České Budějovice, Czech Republic; 13 Canadian Institute for Advanced Research, Botany Department, University of British Columbia, Vancouver, British Columbia, Canada; 14 Faculty of Science, University of South Bohemia, České Budějovice, Czech Republic; 15 Department of Invertebrate Zoology, St-Petersburg State University, St-Petersburg, Russia; 16 Royal Botanic Garden Edinburgh, Edinburgh, United Kingdom; 17 Allgemeine Ökologie, Universität zu Köln, Köln, Germany; 18 Department of Systematic Biology, Evolutionary Biology Centre, Uppsala University, Uppsala, Sweden; 19 Department of Biology, University of New Brunswick, Fredericton, New Brunswick, Canada; 20 Department of Biology, Life Sciences Centre, Halifax, Nova Scotia, Canada; 21 National Center for Biotechnology Information, National Library of Medicine, National Institutes of Health, Computational Biology Branch, Bethesda, Maryland, United States of America; 22 Sir Alister Hardy Foundation for Ocean Science, Citadel Hill, Plymouth, United Kingdom; 23 Justus-Liebig-University, Giessen, Germany; 24 Smithsonian Institution, National Museum of Natural History, Washington, DC, United States of America

## Abstract

A group of protist experts proposes a two-step DNA barcoding approach, comprising a universal eukaryotic pre-barcode followed by group-specific barcodes, to unveil the hidden biodiversity of microbial eukaryotes.

Animals, plants, and fungi—the three traditional kingdoms of multicellular eukaryotic life—make up almost all of the visible biosphere, and they account for the majority of catalogued species on Earth [1]. The remaining eukaryotes have been assembled for convenience into the *protists*, a group composed of many diverse lineages, single-celled for the most part, that diverged after Archaea and Bacteria evolved but before plants, animals, or fungi appeared on Earth. Given their single-celled nature, discovering and describing new species has been difficult, and many protistan lineages contain a relatively small number of formally described species (Figure 1A), despite the critical importance of several groups as pathogens, environmental quality indicators, and markers of past environmental changes. It would seem natural to apply molecular techniques such as DNA barcoding to the taxonomy of protists to compensate for the lack of diagnostic morphological features, but this has been hampered by the extreme diversity within the group. The genetic divergence observed between and within major protistan groups greatly exceeds that found in each of the three multicellular kingdoms. No single set of molecular markers has been identified that will work in all lineages, but an international working group is now close to a solution. A universal DNA barcode for protists coupled with group-specific barcodes will enable an explosion of taxonomic research that will catalyze diverse applications.

**Figure 1:**
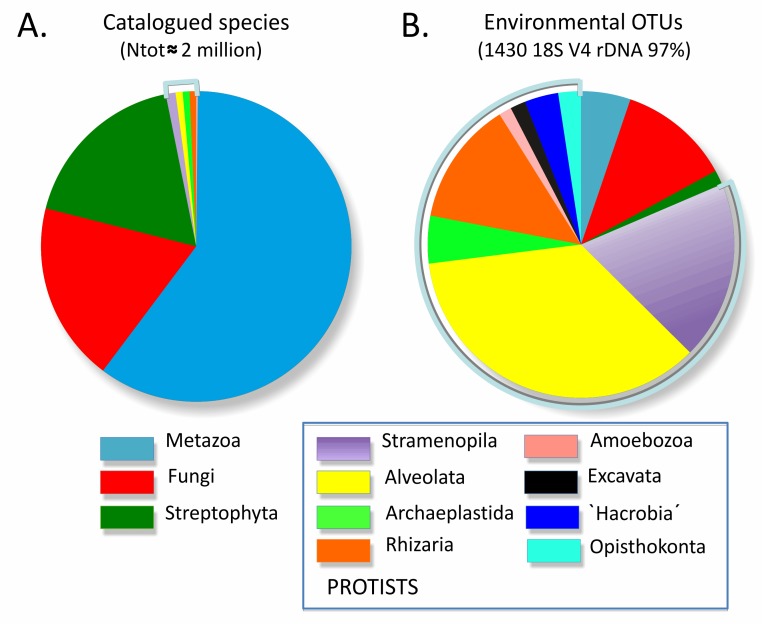
Morphological versus genetic views of total eukaryotic diversity. (A) Relative numbers of described species per eukaryotic supergroup—see Table S1 for a detailed count per division/class. (B) Relative number of V4 18S rDNA Operational Taxonomic Units (97%) per eukaryotic supergroup, based on 59 rDNA clone library surveys of marine, fresh-water, and terrestrial total eukaryotic biodiversity (as listed in [55]).

The undiscovered species diversity among protists may be orders of magnitude greater than previously thought. Surveys of protistan environmental diversity usually based on Sanger sequencing of polymerase chain reaction-amplified 18S rDNA clone libraries revealed an extremely high proportion of sequences that could not be assigned to any described species and in some cases even suggested the presence of several new eukaryotic kingdoms [2],[3]. Although some of these sequences have since been shown to be chimeric or long-branch attraction artefacts (caused by heterogeneity of evolutionary rates) [4], novel protistan phyla continue to be discovered (e.g., [5],[6]). More recently, the growing number of Next Generation Sequencing (NGS) studies of eukaryotic diversity [6]–[9] has confirmed that the evolutionary and ecological importance of protists is much higher than traditionally thought (Figure 1B) and suggest that the number of protist species may easily exceed one million, although the correct estimation depends on many factors discussed below. The flow of eukaryotic sequence data produced by NGS from environmental DNA extracts is exponentially increasing, but there is currently no way to interpret these sequences in terms of species diversity and ecology.

DNA barcoding is a technique that uses a short standardized DNA region to identify species [10]. Large public reference libraries of DNA barcodes are being developed for animals, plants, and fungi, but there is no general agreement on which region to use for protists. Identifying the standard barcode regions for protists and assembling a reference library are the main objectives of the Protist Working Group (ProWG), initiated by the Consortium for the Barcode of Life (CBOL, http://www.barcodeoflife.org/). The ProWG unites a panel of international experts in protist taxonomy and ecology, with the aim to assess and unify the efforts to identify the barcode regions across all protist lineages, create an integrated plan to finalize the selection, and launch projects that would populate the reference barcode library. Here, we discuss the advantages and limitations of DNA barcodes currently in use and introduce a two-steps barcoding approach to assess protistan biodiversity.

## The Unknown Vastness of Protist Richness

**Figure 2:**
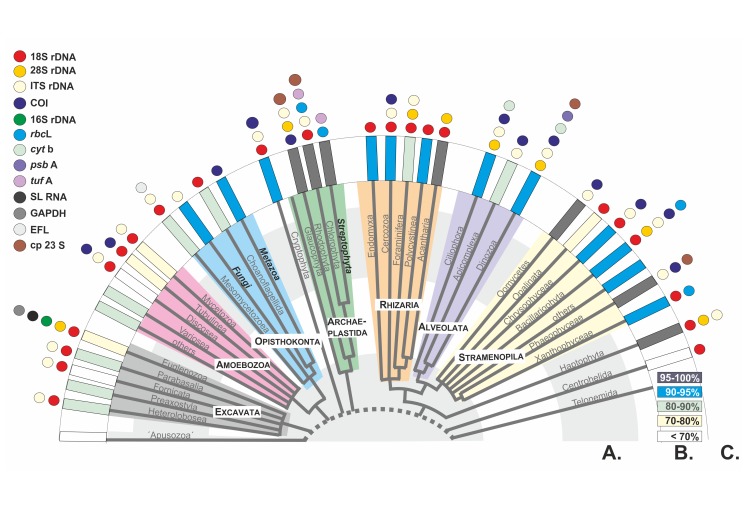
Current state-of-the art phylogeny and barcode markers for the main protistan lineages. (A) A recent phylogeny of eukaryotic life, after [56]. (B) Mean V4 18S rDNA genetic similarity between all congeneric species within each lineage, available in GenBank. (C) Currently used group-specific barcodes. The dashed line indicates the incertitude concerning the position of the root in the tree of eukaryotic life. The unresolved relationships between eukaryotic groups are indicated by polytomies. The names of the three multicellular classical “kingdoms” are highlighted.

The first task of the protist barcoding initiative is to assess species richness in all protistan supergroups. In historically well-studied and biologically well-known taxa, such as higher plants or vertebrates, the number of predicted and described species is relatively similar. The situation is diametrically different for the fungi, for which *catalogued* species comprise ∼7% of the predicted species number [1]. It is even worse for protists. The number of catalogued protistan species is very low in comparison to the diversity of animals, plants, and fungi, ranging from ∼26,010 excluding marine nonphotosynthetic protists [1] to ∼43,000 [11] and ∼74,400 for the novel *ProWG* estimates presented herein (Table S1). Among the seven protistan supergroups (Figure 2A), the most diverse are Stramenopiles, with ∼25,000 morphospecies. Over 10,000 described species are also found in Alveolata, Rhizaria, and Archaeplastida (excluding land-plants). Much fewer species have been catalogued for Amoebozoa (∼2,400), Excavata (∼2,300), and for the unicellular Opisthokonta (∼300)—this latter group being dominated by animals and fungi.

The *predicted* richness of protistan species ranges from 1.4×10^5^ to 1.6×10^6^ [12]. In several groups, the number of predicted species has been arbitrarily estimated to be twice the number of described species [12]. But the true number of species could be several orders of magnitude higher. For example, the Apicomplexa are obligatory parasites, including the malaria agent *Plasmodium* and omnipresent *Toxoplasma*, and thus could reach up to 1.2×10^6^ species if we assume a strict specificity to their metazoan hosts. The same argument can be applied to predict extreme species richness in protistan parasites of fishes (e.g., Mesomycetozoa) and plants (e.g., Oomycetes). However, most of these predictions are highly subjective.

Moreover, just like in Bacteria and Archaea [13],[14], there is no general agreement on how to define species in protists, and no single species concept can be applied unequivocally to all protistan groups. Molecular studies typically reveal a multitude of genotypes hidden within protist species that have been discovered and described using traditional methods based on morphological criteria (often referred to as “morphospecies”). Reproductive isolation could theoretically be used in differentiating eukaryotic species, but data on the very existence of a sexual phase are very sparse in protists. Mating studies in some “model” systems (e.g., [15],[16]) are consistent with the evidence from molecular data that protistan species diversity is greatly underestimated by classical morphological approaches. Overall intraspecific and intragenomic variabilities in environmental protistan populations are still largely unknown, because most genetic studies are carried out on clonal strains maintained in laboratory cultures.

## Protist Barcoding: State of the Art

Although the term *DNA barcoding* appeared only recently in the protistological literature, the identification of protistan taxa using molecular markers has a long history. The most commonly used markers have been parts of the genes coding for ribosomal RNAs, in particular 18S rDNA (e.g., [17]). The advantages of 18S rDNA are many: found in all eukaryotes, it occurs in many copies per genome, allowing genetic work at the individual (single-cell) level; it is highly expressed, permitting molecular ecological investigation at the RNA level; and it includes a mosaic of highly conserved and variable nucleotide sequences allowing combined phylogenetic reconstruction and biota recognition at various taxonomic levels. Different 18S rDNA variable regions have been used in clone libraries and NGS-based environmental surveys [3],[7],[18]. 18S rDNA barcodes have been shown to effectively distinguish species in some groups, such as foraminifera [19],[20] and some diatoms [21], however they are not sufficiently variable to resolve interspecies relationships in several other taxa (Figure 2B).

Various alternative protistan DNA barcodes have been proposed (Figure 2, Table S2). The D1–D2 and/or D2–D3 regions at the 5′ end of 28S rDNA have been positively tested in ciliates [22], haptophytes [23], and acantharians [24] and are also promising for diatoms [25],[26]. Ribosomal internal transcribed spacers (ITS1 and/or ITS2 rDNA), which are the main fungal barcodes [27], are also commonly utilized in oomycetes [28], chlorarachniophytes [29], and green algae [30] and have also been suggested for dinoflagellates [31],[32] and diatoms [33] with some reserve [34]. The mitochondrial gene coding for cytochrome oxidase 1 (COI), which has been proposed as the universal barcode for animals [10], also allows morpho-species identification in red [35]–[37] and brown [38],[39] algae, dinoflagellates [40], some raphid diatoms [41], Euglyphida [42], lobose naked [43] and shelled [44] amoebae, coccolithophorid haptophytes [45], and some ciliates [46],[47]. Other group-specific barcodes include the large subunit of the ribulose-1,5-biphosphate carboxylase–oxygenase gene (*rbc*L) and the chloroplastic 23S rRNA gene for photosynthetic protists [25],[48]–[50], and Spliced Leader RNA genes for trypanosomatids [51]. Clearly, the choice of group-specific barcodes is often a question of tradition or ease of use, and studies systematically comparing the resolution power of different protistan DNA barcodes are rare [25],[42],[43],[52].

## ProWG Objectives and Perspectives

The ultimate objective of the CBOL ProWG is to establish universal criteria for barcode-based species identification in protists. The DNA barcoding approach has several well-known limitations related to the standardization of species identification [53],[54], and addressing some of the challenges raised by genetic identification of protists will certainly require more fundamental research on protistan speciation. The ProWG will organise workshops and seminaries that will provide opportunity to discuss general questions concerning species definition, genetic variations, and applications of DNA barcodes in all protistan groups.

From a practical perspective, the ProWG mission is to establish the genetic standards that will allow recognition of protistan taxa exclusively on the basis of DNA sequence data. Our goal is not to exclude morphological identification but to propose alternative tools that will be more efficient in dealing with the immense protistan biodiversity and more objective and accessible to nonspecialists. In most protistan groups, morphological characters are unreliable for identification at the species level but do provide guides for higher level taxonomic assignments, as well as valuable information about the biology, ecology, and evolution of organisms. Therefore, every protistan reference DNA barcode must be associated with voucher material and/or illustrations providing phenotypic data from the barcoded specimen.

**Figure 3:**
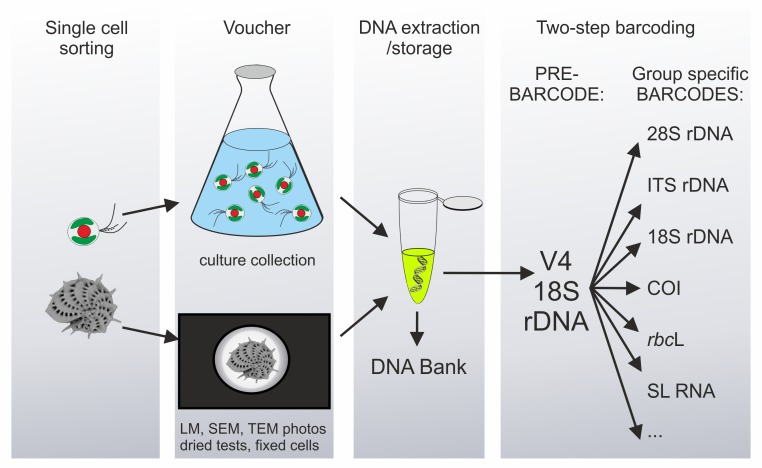
Two-step protist barcoding pipeline. Protistan species, spanning four orders of cell-size magnitude (from <1 µm to >10,000 µm), are individually sorted from the environment, phenotyped either directly or after culture growth, DNA extracted, and barcoded using a two-step, nested strategy.

Because of their long, independent, and complex evolutionary histories, protists are so genetically variable that it is virtually impossible to find a single universal DNA barcode suitable for all of them. The ProWG consortium therefore recommends a two-step barcoding approach, comprising a preliminary identification using a universal eukaryotic barcode, called the pre-barcode, followed by a species-level assignment using a group-specific barcode (Figure 3). In this nested strategy, the ∼500 bp variable V4 region of 18S rDNA is proposed as the universal eukaryotic pre-barcode. Group-specific barcodes (Figure 2C) will then have to be defined separately for each major protistan group, based on comparative studies using the CBOL selection criteria, and much of this work is still to be done. Depending on the type of material (isolates and cultures) and whether or not DNA extraction is destructive for the analysed species, the morphological appearance of each barcoded protist will be preserved as microphotographs, fixed cells, or live and/or cryopreserved cultures. This voucher would be deposited in a public collection, just as type specimens are required for new taxa by the nomenclatural codes. Collection details including locality, date, and (as far as possible) habitat characteristics must also be provided, accompanied in parasitic and symbiotic taxa by an accurately identified host voucher or its DNA/tissue sample wherever this is available. Moreover, the extracted DNA must be deposited in a recognized DNA bank or museum collection and cited with a unique identifier to allow checks and further genetic analyses.

Most of these recommendations are already followed where newly described protistan species are based on cultured strains deposited in collections. However, the large majority of protists are currently uncultivable by known means or not available in culture collections, and genetic data only exist for a very small fraction of described species. Therefore, it is imperative to establish standard barcoding protocols for future protist barcoding projects that will substantially increase the number of collected, described, but uncultivable protists. A combination of novel high-throughput imaging/sorting with newer genetic technologies—including single-amplified-genome methods—opens exciting avenues in protistan metabarcoding. A protist barcoding protocol such as that outlined in Figure 3 will allow collection of the data necessary to set up a representative protist species reference library. The protocols and recommendations concerning protist barcoding will be available at the *ProWG* website (under construction at www.protistbarcoding.org), and a platform dedicated to protist multi-locus barcodes will be accessible at the Barcode of Life Database.

Given the ongoing DNA sequencing revolution, the 21st-century exploration of biodiversity must do more than document the higher macrofaunal and macrofloral branches on the Tree of Life. Amongst other microbes, protists are key but poorly known elements of the ecosystems we see in Nature, including the complex microbiomes hidden within individual plants, animals, and fungi. Ecological models must include protists based on the new knowledge of their species-level diversity that will mostly come from the billions of NGS-generated environmental barcodes. The reference library of standard protistan barcodes will be the Rosetta stone that makes protist diversity less anonymous.

## Supporting Information

Table S1 Number of catalogued morphospecies and V4 18S rDNA OTU-97% among the 60 main eukaryotic lineages.
(PDF)

Table S2 Group-specific barcodes for selected genera representing all eukaryotic supergroups (in brackets, number of corresponding sequences in the GenBank). NM, nucleomorph origin. Variable regions used in 18S and 28S genes are indicated in some cases.
(PDF)
