# Supplementary material for: CBOL Protist Working Group: Barcoding Eukaryotic Richness beyond the Animal, Plant, and Fungal Kingdoms
Source: PLoS Biol. 2012 Nov 6;10(11):e1001419. doi: 10.1371/journal.pbio.1001419 (PMC3491025; doi:10.1371/journal.pbio.1001419)
Supplement: Table S1 — Number of catalogued morphospecies and V4 18S rDNA OTU-97% among the 60 main eukaryotic lineages. (PDF) [file pbio.1001419.s001.pdf]

Supplementary table 1. Number of catalogued morphospecies and V4 18S rDNA OTU-97% amongst the 60 main eukaryotic lineages.

Supergroup	Division/Phylum	Catalogued morpho-species	OTU (V4 rDNA, 97%)*
ALVEOLATA	Apicomplexa	6000	626
	Ciliophora	8000	1157
	Dinophyceae	2280	426
	Ellobiopsidae	6	2
	Perkinsea	0	28
	Syndiniales	0	391
	Chromera	1	1
AMOEBOZOA	Tubulinea + Arcellinida	1100	58
	Discosea	180	77
	Variosea	20	28
	Lobosa incertae sedis	0	60
	Protostelia	32	
	Dictyostelia + Myxogastria	1062	107
	Breviatea	12	13
ARCHAEPLASTIDA	Chlorophyta (- Streptophyta)	9000	740
	Streptophyta	350000	1447
	Glaucocystophyta	5	4
	Rhodophyta	5000	640
EXCAVATA	Euglenozoa	1520	296
	Fornicata	146	45
	Heterolobosea	80	640
	Jakobida	10	13
	Malawimonadidae	3	1
	Parabasalia	466	180
	Preaxostyla	96	22
OPISTHOKONTA	Choanoflagellida	250	88
	Fungi	377200	3694
	Mesomycetozoea	47	56
	Metazoa	1200000	8750
RHIZARIA	Cercozoa	600	749
	Foraminifera	12000	36
	Acantharea	160	36
	Polycystinea	850	91
	other Radiolaria	1	39
STRAMENOPILES	Bacillariophyta	20000	603
	Bicoecea	72	80
	Bolidophyceae and relatives	0	15
	Chrysophyceae-Synurophyceae	1200	273
	Dictyochophyceae	15	45
	Eustigmatophyceae	15	12
	Hyphochytriomyceta	25	3
	Labyrinthulea	40	186
	MAST	0	94
	Oomycota	676	66
	Opalinata	400	53
	Pelagophyceae	12	22
	Phaeophyceae	1750	49
	Phaeothamniophyceae	25	4
	Picophagea	0	0
	Pinguiophyceae	5	7
	Pirsonia	0	7
	Raphidophyceae	20	10
	Xanthophyceae	600	28
INCERTAE SEDIS	Apusomonadidae	12	24
	Centroheliozoa	150	71
	Cryptophyta	70	90

	Haptophyta	350	127
	Hilomonadea	0	7
	Katablepharidophyta	9	15
	Picobiliphyta	0	12
	Telonemia	0	31

* OTU-97% found in the *Genbank nt* and *env_nt* divisions of Genbank (jan 2012). Total of 86,587 sequences.
