# Supplementary material for: CBOL Protist Working Group: Barcoding Eukaryotic Richness beyond the Animal, Plant, and Fungal Kingdoms
Source: PLoS Biol. 2012 Nov 6;10(11):e1001419. doi: 10.1371/journal.pbio.1001419 (PMC3491025; doi:10.1371/journal.pbio.1001419)
Supplement: Table S2 — Group-specific barcodes for selected genera representing all eukaryotic supergroups (in brackets, number of corresponding sequences in the GenBank). NM, nucleomorph origin. Variable regions used in 18S and 28S genes are indicated in some cases. (PDF) [file pbio.1001419.s002.pdf]

Supplementary Table 2. Group-specific barcodes for selected genera representing all eukaryotic supergroups (in brackets number of corresponding sequences in the GenBank). NM denotes nucleomorph origin. Variable regions used in 18S and 28S genes are indicated in some cases.

Supergroup	Phylum/Class	Genus	Barcode 1	Barcode 2	Barcode 3	References
ALVEOLATA	Apicomplexa	Eimeria	ITS (542)	COI (102)		[1,2]
		Neospora	ITS1 (75)			[3,4]
		Sarcocystis	ITS1 (144)	COI (9)		[5]
	Ciliophora	Carchesium	ITS (62)	COI (59)	28S (58)	[6]
		Coleps	cyt b (28)			[7]
		Paramecium	ITS (388)	COI (210)		[8], [9], [10]
		Tetrahymena	COI (141)	18S (34)		[11-13]
	Dinophyceae	Alexandrium	ITS (452)	28S/D1-D2 (220)	COI (66) cob (5)	[14-17]
		Pfiesteria	ITS (28)	18S (11)	COI (8)	[17,18]
		Prorocentrum	COI (74)	ITS (24)	cob (7)	[15-19]
		Symbiodinium	ITS 1 (73) ITS2 (2551)	psbA (97)	Cp 23S (592)	[16,17,20-23]
		metabarcoding	COI	cytb		[17]
	Perkinsea	Perkinsus	ITS (387)	NTS (23)		[24,25]
	Syndiniales	Euduboscquella	ITS (62)			[26]
AMOEBOZOA	Archamoebae	Entamoeba	18S (123)	ITS (17)		[27,28]
	Centramoebae	Acanthamoeba	18S (1240)			[29-32]
	Discosea	Cochliopodium	18S (16)	COI (2)		[33,34]
		Parvamoeba	18S (7)	COI (2)		[35]
		Paramoeba	18S (5)			[36]
		Neoparamoeba	ITS (154)	18S (33)		[37-39]
		Vannella	COI (236)	ITS (109)		[40]
	Dictyostelia	various genera	ITS (364)	18S (169)		[41,42]
	Myxogastria	various genera	18S/5-11 (101)	ITS (59)		[43-46]
	Protostelia	various genera	18S (28)			[43,47]
	Tubulinea/Arcellinida	various genera	COI (59)	18S (18)		[48-50]
ARCHAEPLASTIDA	Chlorophyta	Caulerpa	ITS2 (450)	tufA (383)		[51,52]
		Volvocales	ITS2 (234)	18S (735)		[53]
		Ulva	ITS2 (621)	rbcL (81)	tufA (138) Cp 23S (3)	[54,55]
		various genera	Cp 23S (16)			[55]
	Rhodophyta	various genera	COI (8767)	ITS (1516)	rbcL (5353) 28S (1649)	[56-61]
EXCAVATA	Euglenozoa/Euglenida	various genera	18S (287)	28S (289)	16S (242)	[62]
	Euglenozoa/Kinetoplastea	Bodo/Neobodo	18S (87)			[62-64]
	Euglenozoa/Kinetoplastea	Trypanosomatidae	SL RNA	18S (880)	GAPDH (90)	[65-68]

			(939)			
	Fornicata	various genera	18S (148)	ITS (89)		[69]
	Heterolobosea	Naegleria	ITS (322)	18S (58)		[70,71]
	Parabasalia	various genera	ITS (343)	18S (73)		[72-75]
**OPISTHOKONTA**	Choanoflagellida	various genera	18S/V4 (68)			[76]
	Mesomycetozoea/Ichtyosporea	various genera	ITS (475)	18S (148)	EFL (62)	[77,78]
**RHIZARIA**	Cercozoa/Euglyphida	various genera	COI (35)	18S (14)		[50,79-82]
	Cercozoa/Glissomonadida	various genera	18S (164)			[83,84]
	Cercozoa/Chlorarachniophyceae	various genera	ITSnm (19)	ITSnuc (10)		[85]
	Cercozoa	various genera	18S (465)	ITS (154)		[85-88]
	Plasmodiophorida	various genera	ITS (85)			[89-91]
	Haplosporidia	Bonamia	ITS (132)	18S (19)	Actin1 (6)	[92,93]
	Foraminifera	various genera	18S/32-50 (2147)	28S/D1-D2 (331)	ITS (659)	[94-96]
	Radiolaria/Acantharea	various genera	28S (113)	18S (88)		[97]
	Radiolaria/Polycystinea	various genera	18S (46)	ITS (10)		[98,99]
**STRAMENOPILES**	Bacillariophyta	Sellaphora, some raphid pennates	rbcL (105)	COI (59)	28S/D2-D3 (79)	[100-105]
		various genera	18S / V4 (3039)	ITS (1204)	rbcL (105)	[102,106-108]
	Bicoecea	various genera	18S (58)	28S (12)		[82]
	Blastocystis	Blastocystis	18S (291)			[109-112]
	Bolidophyceae	various genera	18S (11)			[113]
	Chrysophyceae-Synurophyceae	Dinobryon	COI (44)	ITS (44)		[114]
		Synura	ITS (139)	18S (113)		[115]
	Dictyochophyceae	various genera	18S (33)	ITS (46)		[116,117]
	Eustigmatophyceae	various genera	18S (96)	ITS (8)		[117]
	Labyrinthulea	various genera	18S (435)	ITS (85)		[118]
	Oomyceta	various genera	ITS (7029)	COI (450)	18S (456)	[81,119]
	Slopalinida	various genera	18S (14)			[120,121]
	Pelagophyceae	various genera	18S (64)			[117]
	Phaeophyceae	Fucus	COI (125)	ITS (116)		[122]
	Phaeophyceae	various genera	COI (1781)	ITS (1459)	Cp 23S (14)	[55,123-125]
	Raphidophyceae	Chattonella	COI (156)	ITS (119)	18S (20)	[126]
	Xanthophyceae	various genera	rbcL (266)	18S (73)		[127]
**XXX**	Centroheliozoa	various genera	18S (40)			[128]
**XXX**	Cryptophyta	various genera	18S (299)	ITS (66)		[129]
**XXX**	Haptophyta	various genera	18S (338)	28S (113)	ITS (45)	[130]

References

1. Motriuk-Smith D, Seville RS, Quealy L, Oliver CE (2011) Comparison of the ITS1 and ITS2 rDNA in Eimeria callospermophili (Apicomplexa:Eimeriidae) from sciurid rodents. J Parasitol 97: 305-310.
2. Ogedengbe JD, Hanner RH, Barta JR (2011) DNA barcoding identifies Eimeria species and contributes to the phylogenetics of coccidian parasites (Eimeriorina, Apicomplexa, Alveolata). Int J Parasitol 41: 843-850.
3. Gondim LF, Laski P, Gao L, McAllister MM (2004) Variation of the internal transcribed spacer 1 sequence within individual strains and among different strains of Neospora caninum. J Parasitol 90: 119-122.
4. Slapeta JR, Koudela B, Votypka J, Modry D, Horejs R, et al. (2002) Coprodiagnosis of Hammondia heydorni in dogs by PCR based amplification of ITS 1 rRNA: differentiation from morphologically indistinguishable oocysts of Neospora caninum. Vet J 163: 147-154.
5. Wendte JM, Miller MA, Nandra AK, Peat SM, Crosbie PR, et al. (2010) Limited genetic diversity among Sarcocystis neurona strains infecting southern sea otters precludes distinction between marine and terrestrial isolates. Vet Parasitol 169: 37-44.
6. Gentekaki E, Lynn DH (2009) High-level genetic diversity but no population structure inferred from nuclear and mitochondrial markers of the peritrichous ciliate Carchesium polypinum in the Grand River basin (North America). Appl Environ Microbiol 75: 3187-3195.
7. Barth D, Tischer K, Berger H, Schlegel M, Berendonk TU (2008) High mitochondrial haplotype diversity of Coleps sp. (Ciliophora: Prostomatida). Environ Microbiol 10: 626-634.
8. Barth D, Krenek S, Fokin SI, Berendonk TU (2006) Intraspecific genetic variation in Paramecium revealed by mitochondrial cytochrome C oxidase I sequences. J Eukaryot Microbiol 53: 20-25.
9. Struder-Kypke MC LD (2010) Comparative analysis of the mitochondrial cytochrome c oxidase subunit I (COI) gene in ciliates (Alveolata, Ciliophora) and evaluation of its suitability as a biodiversity marker. Systematics and Biodiversity 8:131-148.
10. Greczek-Stachura M, Potekhin A, Przybos E, Rautian M, Skoblo I, et al. (2011) Identification of Paramecium bursaria Syngens through Molecular Markers - Comparative Analysis of Three Loci in the Nuclear and Mitochondrial DNA. Protist.
11. Chantangsi C, Lynn DH, Brandl MT, Cole JC, Hetrick N, et al. (2007) Barcoding ciliates: a comprehensive study of 75 isolates of the genus Tetrahymena. Int J Syst Evol Microbiol 57: 2412-2425.
12. Kher CP, Doerder FP, Cooper J, Ikonomi P, Achilles-Day U, et al. (2011) Barcoding Tetrahymena: discriminating species and identifying unknowns using the cytochrome c oxidase subunit I (cox-1) barcode. Protist 162: 2-13.
13. Lynn DH, Struder-Kypke MC (2006) Species of tetrahymena identical by small subunit rRNA gene sequences are discriminated by mitochondrial cytochrome c oxidase I gene sequences. J Eukaryot Microbiol 53: 385-387.
14. Kim CJ, Kim CH, Sako Y (2005) Development of molecular identification method for genus Alexandrium (Dinophyceae) using whole-cell FISH. Mar Biotechnol (NY) 7: 215-222.
15. Lin S, Zhang H, Hou Y, Zhuang Y, Miranda L (2009) High-level diversity of dinoflagellates in the natural environment, revealed by assessment of mitochondrial cox1 and cob genes for dinoflagellate DNA barcoding. Appl Environ Microbiol 75: 1279-1290.
16. Stern RF, Andersen, R.A., Jameson, I., Küpper, F.C., Coffroth, M.A., Vaulot, D., Le Gall, F., Véron, B., Brand, J.J., Skelton, H., Kasai, F., Lilly, E.L., Keeling, P.J. (2012) Evaluating the ribosomal internal transcribed spacer (ITS) as a candidate dinoflagellate barcode marker. . PLoS ONE [pas encore sorti].
17. Stern RF, Horak A, Andrew RL, Coffroth MA, Andersen RA, et al. (2010) Environmental barcoding reveals massive dinoflagellate diversity in marine environments. PLoS One 5: e13991.
18. Litaker RW VM, Kibler SR, Reece KS, Stokes NA, Lutzoni FM, Yonish BA, West MA, Black MND, Tester PA (2007) Recognizing Dinoflagellate species using its rDNA sequences. J Phycol 43: 344–355.
19. Ferrell J (2008) The evaluation of DNA barcoding for species identification of dinoflagellates,. Mount Allison University.
20. LaJeunesse TC, Thornhill DJ (2011) Improved resolution of reef-coral endosymbiont (Symbiodinium) species diversity, ecology, and evolution through psbA non-coding region genotyping. PLoS One 6: e29013.
21. Pochon X, Putnam HM, Burki F, Gates RD (2012) Identifying and characterizing alternative molecular markers for the symbiotic and free-living dinoflagellate genus Symbiodinium. PLoS One 7: e29816.

22. Pochon X LT, Pawlowski J. (2004) Biogeographic partitioning and host specialization among foraminiferan dinoflagellate symbionts (Symbiodinium ; Dinophyta) Marine Biology 146: 17–27.
23. Santos S, Gutierrez-Rodriquez, C, Coffroth, MA (2003) Phylogenetic identification of symbiotic dinoflagellates via length heteroplasmy in domain V of chloroplast large subunit (cp23S)—ribosomal DNA sequences. Mar Biotechnol (NY) 5(2): 130-140.
24. Elandaloussi L, Carrasco N, Furones D, Roque A (2009) Phylogenetic relationship of Perkinsus olseni from the Ebro Delta, Spain, to other Perkinsus species, based on ribosomal DNA sequences. Dis Aquat Organ 86: 135-142.
25. Park KI, Park JK, Lee J, Choi KS (2005) Use of molecular markers for species identification of Korean Perkinsus sp. isolated from Manila clams Ruditapes philippinarum. Dis Aquat Organ 66: 255-263.
26. Bachvaroff TR, Kim S, Guillou L, Delwiche CF, Coats DW (2012) Molecular diversity of the syndinean genus Euduboscquella based on single-cell PCR analysis. Appl Environ Microbiol 78: 334-345.
27. Stensvold CR, Lebbad M, Verweij JJ, Jespersgaard C, von Samson-Himmelstjerna G, et al. (2010) Identification and delineation of members of the Entamoeba complex by pyrosequencing. Mol Cell Probes 24: 403-406.
28. Stensvold CR, Lebbad M, Victory EL, Verweij JJ, Tannich E, et al. (2011) Increased sampling reveals novel lineages of Entamoeba: consequences of genetic diversity and host specificity for taxonomy and molecular detection. Protist 162: 525-541.
29. Booton GC, Visvesvara GS, Byers TJ, Kelly DJ, Fuerst PA (2005) Identification and distribution of Acanthamoeba species genotypes associated with nonkeratitis infections. J Clin Microbiol 43: 1689-1693.
30. Corsaro DV, D. (2011) More Acanthamoeba genotypes: limits to the use of rDNA fragments to describe new genotypes. . Acta Protozool, 50:49-54.
31. Ledee DR, Iovieno A, Miller D, Mandal N, Diaz M, et al. (2009) Molecular identification of t4 and t5 genotypes in isolates from acanthamoeba keratitis patients. J Clin Microbiol 47: 1458-1462.
32. Stothard DR, Schroeder-Diedrich JM, Awwad MH, Gast RJ, Ledee DR, et al. (1998) The evolutionary history of the genus Acanthamoeba and the identification of eight new 18S rRNA gene sequence types. J Eukaryot Microbiol 45: 45-54.
33. Kudryavtsev A, Bernhard D, Schlegel M, Chao EE, Cavalier-Smith T (2005) 18S ribosomal RNA gene sequences of Cochliopodium (Himatismenida) and the phylogeny of Amoebozoa. Protist 156: 215-224.
34. Kudryavtsev A. WK, Pawlowski J. (2011) Ovalopodium desertum n. sp. and the phylogenetic relationships of Cochliopodiidae (Amoebozoa). Protist 162:571-589.
35. Kudryavtsev A (2012) Microscopic evidence for inclusion of Parvamoeba Rogerson, 1993 into the order Himatismenida (Amoebozoa). Europ J Protistol 47:85-88.
36. Kudryavtsev A PJ, Hausmann K (2011) Description of Paramoeba atlantica n. sp. (Amoebozoa, Dactylopodida) – a marine amoeba from the Eastern Atlantic, with emendation of the dactylopodid families. Acta Protozool 50:239-253.
37. Dykova I, Nowak B, Peckova H, Fiala I, Crosbie P, et al. (2007) Phylogeny of Neoparamoeba strains isolated from marine fish and invertebrates as inferred from SSU rDNA sequences. Dis Aquat Organ 74: 57-65.
38. Dykova I, Nowak BF, Crosbie PB, Fiala I, Peckova H, et al. (2005) Neoparamoeba branchiphila n. sp., and related species of the genus Neoparamoeba Page, 1987: morphological and molecular characterization of selected strains. J Fish Dis 28: 49-64.
39. Fiala I, Dykova I (2003) Molecular characterisation of Neoparamoeba strains isolated from gills of Scophthalmus maximus. Dis Aquat Organ 55: 11-16.
40. Nassonova E, Smirnov A, Fahrni J, Pawlowski J (2010) Barcoding amoebae: comparison of SSU, ITS and COI genes as tools for molecular identification of naked lobose amoebae. Protist 161: 102-115.
41. Romeralo M, Cavender JC, Landolt JC, Stephenson SL, Baldauf SL (2011) An expanded phylogeny of social amoebas (Dictyostelia) shows increasing diversity and new morphological patterns. BMC Evol Biol 11: 84.
42. Romeralo M, Escalante R, Sastre L, Lado C (2007) Molecular systematics of dictyostelids: 5.8S ribosomal DNA and internal transcribed spacer region analyses. Eukaryot Cell 6: 110-116.
43. Fiore-Donno AM, Kamono A, Chao EE, Fukui M, Cavalier-Smith T (2010) Invalidation of Hyperamoeba by transferring its species to other genera of Myxogastria. J Eukaryot Microbiol 57: 189-196.
44. Fiore-Donno AM, Meyer M, Baldauf SL, Pawlowski J (2008) Evolution of dark-spored Myxomycetes (slime-molds): molecules versus morphology. Mol Phylogenet Evol 46: 878-889.
45. Fiore-Donno AM, Novozhilov YK, Meyer M, Schnittler M (2011) Genetic structure of two protist species (Myxogastria, Amoebozoa) suggests asexual reproduction in sexual Amoebae. PLoS One 6: e22872.

46. Winsett KE SS (2008) Using ITS sequences to assess intraspecific genetic relationships among geographically separated collections of the myxomycete Didymium squamulosum. Rev Mex Micol 27:59–65.
47. Shadwick LL, Spiegel FW, Shadwick JD, Brown MW, Silberman JD (2009) Eumycetozoa = Amoebozoa?: SSUrDNA phylogeny of protosteloid slime molds and its significance for the amoebozoan supergroup. PLoS One 4: e6754.
48. Kosakyan A, Heger TJ, Leander BS, Todorov M, Mitchell EA, et al. (2012) COI Barcoding of Nebelid Testate Amoebae (Amoebozoa: Arcellinida): Extensive Cryptic Diversity and Redefinition of the Hyalospheniidae Schultze. Protist 163: 415-434.
49. Kudryavtsev A, Pawlowski J, Hausmann K (2009) Description and phylogenetic relationships of Spumochlamys perforata n. sp. and Spumochlamys bryora n. sp. (Amoebozoa, Arcellinida). J Eukaryot Microbiol 56: 495-503.
50. Lara E, Heger TJ, Ekelund F, Lamentowicz M, Mitchell EA (2008) Ribosomal RNA genes challenge the monophyly of the Hyalospheniidae (Amoebozoa: Arcellinida). Protist 159: 165-176.
51. Jousson O, Pawlowski J, Zaninetti L, Zechman FW, Dini F, et al. (2000) Invasive alga reaches California. Nature 408: 157-158.
52. Stam WT, Olsen, J.L., Zaleski, S.F., Murray, S.N., Brown, K.R. & Walters, L.J. (2006) A forensic and phylogenetic survey of Caulerpa species (Caulerpales, Chlorophyta) from the Florida coast, local aquarium shops, and e-commerce: establishing a proactive baseline for early detection. Journal of Phycology 42: 1113-1124.
53. Coleman AW (2000) The significance of a coincidence between evolutionary landmarks found in mating affinity and a DNA sequence. Protist 151: 1-9.
54. Saunders GV KH (2010) An evaluation of rbcL, tufA, UPA, LSU and ITS as DNA barcode markers for the marine green macroalgae. Cryptogamie, Algologie, 31: 487-528.
55. Sherwood AR, Prestling, G.G. (2007) Universal primers amplify a 23S rDNA plastid marker in eukaryotic algae and cyanobacteria. J Phycol 43(3): 605-608.
56. Clarkston BES, G.W. (2010) A comparison of two DNA barcode markers for species discrimination in the red algal family Kallymeniaceae (Gigartinales), with a description of Euthora timburtonii. Botany 88: 119-131.
57. Le Gall LS, G.W. (2010) DNA barcoding is a powerful tool to uncover algal diversity: a case study of the Phyllophoraceae (Gigartinales, Rhodophyta) in the Canadian flora. J Phycol 46: 374-389.
58. Robba L, Russell SJ, Barker GL, Brodie J (2006) Assessing the use of the mitochondrial cox1 marker for use in DNA barcoding of red algae (Rhodophyta). Am J Bot 93: 1101-1108.
59. Saunders GW (2005) Applying DNA barcoding to red macroalgae: a preliminary appraisal holds promise for future applications. Philos Trans R Soc Lond B Biol Sci 360:1879-88.
60. Saunders GW (2008) A DNA barcode examination of the red algal family Dumontiaceae in Canadian waters reveals substantial cryptic species diversity. 1. The foliose Dilsea-Neodilsea complex and Weeksia. Botany 86: 773-789 (shared cover article).
61. Saunders GW (2009) Routine DNA barcoding of Canadian Gracilariales (Rhodophyta) reveals the invasive species Gracilaria vermiculophylla in British Columbia. Molecular Ecology Resources 9 (Suppl 1), 140–150
62. Linton EW, Karnkowska-Ishikawa A, Kim JI, Shin W, Bennett MS, et al. (2010) Reconstructing euglenoid evolutionary relationships using three genes: nuclear SSU and LSU, and chloroplast SSU rDNA sequences and the description of Euglenaria gen. nov. (Euglenophyta). Protist 161: 603-619.
63. von der Heyden S, Cavalier-Smith T (2005) Culturing and environmental DNA sequencing uncover hidden kinetoplastid biodiversity and a major marine clade within ancestrally freshwater Neobodo designis. Int J Syst Evol Microbiol 55: 2605-2621.
64. von der Heyden S, Chao EE, Vickerman K, Cavalier-Smith T (2004) Ribosomal RNA phylogeny of bodonid and diplonemid flagellates and the evolution of euglenozoa. J Eukaryot Microbiol 51: 402-416.
65. Maslov DA, Westenberger SJ, Xu X, Campbell DA, Sturm NR (2007) Discovery and barcoding by analysis of spliced leader RNA gene sequences of new isolates of Trypanosomatidae from Heteroptera in Costa Rica and Ecuador. J Eukaryot Microbiol 54: 57-65.
66. Votypka J, Maslov DA, Yurchenko V, Jirku M, Kment P, et al. (2010) Probing into the diversity of trypanosomatid flagellates parasitizing insect hosts in South-West China reveals both endemism and global dispersal. Mol Phylogenet Evol 54: 243-253.
67. Yurchenko VY, Lukes J, Jirku M, Zeledon R, Maslov DA (2006) Leptomonas costaricensis sp. n. (Kinetoplastea: Trypanosomatidae), a member of the novel phylogenetic group of insect trypanosomatids closely related to the genus Leishmania. Parasitology 133: 537-546.

68. Yurchenko VY, Lukes J, Tesarova M, Jirku M, Maslov DA (2008) Morphological discordance of the new trypanosomatid species phylogenetically associated with the genus crithidia. Protist 159: 99-114.
69. Kolisko M, Silberman JD, Cepicka I, Yubuki N, Takishita K, et al. (2010) A wide diversity of previously undetected free-living relatives of diplomonads isolated from marine/saline habitats. Environ Microbiol 12: 2700-2710.
70. De Jonckheere JF (2006) Isolation and molecular identification of free-living amoebae of the genus Naegleria from Arctic and sub-Antarctic regions. Eur J Protistol 42: 115-123.
71. Edagawa A, Kimura A, Kawabuchi-Kurata T, Kusuhara Y, Karanis P (2009) Isolation and genotyping of potentially pathogenic Acanthamoeba and Naegleria species from tap-water sources in Osaka, Japan. Parasitol Res 105: 1109-1117.
72. Gile GH, James, E. R., Scheffrahn, R. H., Carpenter, K. J., and Keeling, P. J. (2011) Molecular and morphological analysis of the Calonymphidae with a description of Calonympha chia sp. nov.,Snyderella kirbyi sp. nov., and Snyderella swezyae sp. nov. Int. . J Syst Evol Microbiol 61:2547-58.
73. Harper JT, Gile, G. H., James, E. R., Carpenter, K. J. and Keeling, P. J. (2009) The inadequacy of morphology for species and genus delineation in microbial eukaryotes: an example from the parabasalian termite symbiont Coronympha. PLoS One 4, e6577
74. Saldarriaga JF, Gile, G. H., James, E. R., Horak, A., Scheffrahan, R. H., and Keeling, P. J. (2011) Morphology and molecular phylogeny of Pseudotrichonympha paulistana and Pseudotrichonympha hertwigi (Trichonymphea, Parabasalia) from neotropical rhinotermitids. J Eukaryot Microbiol 58:487-96
75. Smejkalova P, Petrzelkova KJ, Pomajbikova K, Modry D, Cepicka I (2012) Extensive diversity of intestinal trichomonads of non-human primates. Parasitology 139: 92-102.
76. Nitsche F, Carr M, Arndt H, Leadbeater BS (2011) Higher level taxonomy and molecular phylogenetics of the Choanoflagellatea. J Eukaryot Microbiol 58: 452-462.
77. Marshall WL, Berbee ML (2010) Population-level analyses indirectly reveal cryptic sex and life history traits of Pseudoperkinsus tapetis (Ichthyosporea, Opisthokonta): a unicellular relative of the animals. Mol Biol Evol 27: 2014-2026.
78. Marshall WL, Celio G, McLaughlin DJ, Berbee ML (2008) Multiple isolations of a culturable, motile Ichthyosporean (Mesomycetozoa, Opisthokonta), Creolimax fragrantissima n. gen., n. sp., from marine invertebrate digestive tracts. Protist 159: 415-433.
79. Heger TJ, Mitchell EA, Todorov M, Golemansky V, Lara E, et al. (2010) Molecular phylogeny of euglyphid testate amoebae (Cercozoa: Euglyphida) suggests transitions between marine supralittoral and freshwater/terrestrial environments are infrequent. Mol Phylogenet Evol 55: 113-122.
80. Heger TJ, Pawlowski J, Lara E, Leander BS, Todorov M, et al. (2011) Comparing potential COI and SSU rDNA barcodes for assessing the diversity and phylogenetic relationships of cyphoderiid testate amoebae (Rhizaria: Euglyphida). Protist 162: 131-141.
81. Lara E HT, Scheihing R, Mitchell EAD (2011) COI gene and ecological data suggest size-dependent high dispersal and low intra-specific diversity in free-livingterrestrial protists (Euglyphida: Assulina). J Biogeogr 38:640–650.
82. Wylezich C, Nies G, Mylnikov AP, Tautz D, Arndt H (2010) An evaluation of the use of the LSU rRNA D1-D5 domain for DNA-based taxonomy of eukaryotic protists. Protist 161: 342-352.
83. Howe AT, Bass D, Chao EE, Cavalier-Smith T (2011) New genera, species, and improved phylogeny of Glissomonadida (Cercozoa). Protist 162: 710-722.
84. Howe AT, Bass D, Vickerman K, Chao EE, Cavalier-Smith T (2009) Phylogeny, taxonomy, and astounding genetic diversity of glissomonadida ord. nov., the dominant gliding zooflagellates in soil (Protozoa: Cercozoa). Protist 160: 159-189.
85. Gile GH SR, James ER, Keeling PJ (2010) DNA barcoding of Chlorarachniophytes using nucleomorph ITS sequences. J Phycol 46, 743–750.
86. Bass D, Chao EE, Nikolaev S, Yabuki A, Ishida K, et al. (2009) Phylogeny of novel naked Filose and Reticulose Cercozoa: Granofilosea cl. n. and Proteomyxidea revised. Protist 160: 75-109.
87. Bass D, Howe AT, Mylnikov AP, Vickerman K, Chao EE, et al. (2009) Phylogeny and classification of Cercomonadida (Protozoa, Cercozoa): Cercomonas, Eocercomonas, Paracercomonas, and Cavernomonas gen. nov. Protist 160: 483-521.
88. Bass D, Richards TA, Matthai L, Marsh V, Cavalier-Smith T (2007) DNA evidence for global dispersal and probable endemicity of protozoa. BMC Evol Biol 7: 162.

89. Smith MJ, Adams MJ, Ward E (2011) Evidence that Polymyxa species may infect Arabidopsis thaliana. FEMS Microbiol Lett 318: 35-40.
90. Vaianopoulos C, Bragard C, Dieryck B, Moreau V, Maraite H, et al. (2007) A certain but non-exclusive association between Polymyxa graminis special forms and cereals. Commun Agric Appl Biol Sci 72: 745-750.
91. Ward E AM (1998) Analysis of ribosomal DNA sequences of Polymyxa species and related fungi and the development of genus- and species-specific PCR primers. Mycological Research 102:965-974.
92. Cochennec N, Le Roux F, Berthe F, Gerard A (2000) Detection of Bonamia ostreae based on small subunit ribosomal probe. J Invertebr Pathol 76: 26-32.
93. Robert M, Garcia C, Chollet B, Lopez-Flores I, Ferrand S, et al. (2009) Molecular detection and quantification of the protozoan Bonamia ostreae in the flat oyster, Ostrea edulis. Mol Cell Probes 23: 264-271.
94. Holzmann M PJ (1997) Molecular, morphological and ecological evidence for species recognition in Ammonia (Foraminifera). Journal Foraminiferal Research, 27: 311-318.
95. Pawlowski J, Fahrni J, Lecroq B, Longet D, Cornelius N, et al. (2007) Bipolar gene flow in deep-sea benthic foraminifera. Mol Ecol 16: 4089-4096.
96. Pawlowski J, Lecroq B (2010) Short rDNA barcodes for species identification in foraminifera. J Eukaryot Microbiol 57: 197-205.
97. Decelle J, Suzuki N, Mahe F, de Vargas C, Not F (2012) Molecular phylogeny and morphological evolution of the acantharia (radiolaria). Protist 163: 435-450.
98. Krabberod AK, Brate J, Dolven JK, Ose RF, Klaveness D, et al. (2011) Radiolaria divided into Polycystina and Spasmaria in combined 18S and 28S rDNA phylogeny. PLoS One 6: e23526.
99. Kunitomo Y, Sarashina I, Iijima M, Endo K, Sashida K (2006) Molecular phylogeny of acantharian and polycystine radiolarians based on ribosomal DNA sequences, and some comparisons with data from the fossil record. Eur J Protistol 42: 143-153.
100. Evans KM, Chepurnov VA, Mann DG (2009) Ten microsatellite markers for the freshwater diatom Sellaphora capitata. Mol Ecol Resour 9: 216-218.
101. Evans KM, Wortley AH, Mann DG (2007) An assessment of potential diatom "barcode" genes (cox1, rbcL, 18S and ITS rDNA) and their effectiveness in determining relationships in Sellaphora (Bacillariophyta). Protist 158: 349-364.
102. Evans KM MD (2009) A proposed protocol for nomenclaturally effective DNA barcoding of microalgae. Phycologia 48:70–74.
103. Hamsher SE, Evans KM, Mann DG, Poulickova A, Saunders GW (2011) Barcoding diatoms: exploring alternatives to COI-5P. Protist 162: 405-422.
104. Mann DG SS, Trobajo R, Vanormelingen P, Souffreau C. (2010) DNA barcoding for species identification and discovery in diatoms. Cryptogamie: Algologie 31:557–577.
105. Trobajo R MD, Clavero E, Evans KM, Vanormelingen P, McGregor RC (2010) The use of partial cox1, rbcL and LSU rDNA sequences for phylogenetics and species identification within the Nitzschia palea complex (Bacillariophyceae). Eur J Phycol 45:413–425.
106. MacGillivary ML, Kaczmarska I (2011) Survey of the efficacy of a short fragment of the rbcL gene as a supplemental DNA barcode for diatoms. J Eukaryot Microbiol 58: 529-536.
107. Moniz MB, Kaczmarska I (2010) Barcoding of diatoms: nuclear encoded ITS revisited. Protist 161: 7-34.
108. Zimmermann J JR, Gemeinholzer B (2011) Barcoding diatoms: evaluation of the V4 subregion on the 18S rRNA gene, including new primers and protocols. Org Divers Evol 11(3):173-192
109. Scicluna SM, Tawari B, Clark CG (2006) DNA barcoding of blastocystis. Protist 157: 77-85.
110. Stensvold CR, Suresh GK, Tan KS, Thompson RC, Traub RJ, et al. (2007) Terminology for Blastocystis subtypes--a consensus. Trends Parasitol 23: 93-96.
111. Stensvold CR, Traub RJ, von Samson-Himmelstjerna G, Jespersgaard C, Nielsen HV, et al. (2007) Blastocystis: subtyping isolates using pyrosequencing technology. Exp Parasitol 116: 111-119.
112. Tan KS (2008) New insights on classification, identification, and clinical relevance of Blastocystis spp. Clin Microbiol Rev 21: 639-665.
113. Guillou L, Moon-Van Der Staay SY, Claustre H, Partensky F, Vaulot D (1999) Diversity and abundance of Bolidophyceae (Heterokonta) in two oceanic regions. Appl Environ Microbiol 65: 4528-4536.
114. Jost SO MR, Boenigk J (2010) Cultivation-independent species identification of dinobryon species (Chrysophyceae) by means of multiplex single-cell Pcr1. J Phycology 46:901-906.

115. Kynclova AS, P.; Skaloudova, M. (2010) COI gene and ecological data suggest size-dependent high dispersal and low intra-specific diversity in free-livingterrestrial protists (Euglyphida: Assulina) J Biogeogr, 38:640-650.
116. Riisberg I EB (2008) Genetic variation in bloom-forming ichthyotoxic Pseudochattonella species (Dictyochophyceae, Heterokonta) using nuclear, mitochondrial and plastid DNA sequence data. European J Phycol 43: 413-422.
117. Yang EC, Boo GH, Kim HJ, Cho SM, Boo SM, et al. (2012) Supermatrix data highlight the phylogenetic relationships of photosynthetic stramenopiles. Protist 163: 217-231.
118. Bergmann N, Fricke B, Schmidt MC, Tams V, Beining K, et al. (2011) A quantitative real-time polymerase chain reaction assay for the seagrass pathogen Labyrinthula zosterae. Mol Ecol Resour 11: 1076-1081.
119. Robideau GP, De Cock AW, Coffey MD, Voglmayr H, Brouwer H, et al. (2011) DNA barcoding of oomycetes with cytochrome c oxidase subunit I and internal transcribed spacer. Mol Ecol Resour 11: 1002-1011.
120. Kostka M, Cepicka I, Hampl V, Flegr J (2007) Phylogenetic position of Karotomorpha and paraphyly of Proteromonadidae. Mol Phylogenet Evol 43: 1167-1170.
121. Nishi A, Ishida K, Endoh H (2005) Reevaluation of the evolutionary position of opalinids based on 18S rDNA, and alpha- and beta-tubulin gene phylogenies. J Mol Evol 60: 695-705.
122. Kucera ES, G.W. (2008) Assigning morphological variants of Fucus (Fucales, Phaeophyccae) in Canadian waters to recognized species using DNA barcoding. Botany, 86: 1065-1079.
123. Lane CE, Lindstrom SC, Saunders GW (2007) A molecular assessment of northeast Pacific Alaria species (Laminariales, Phaeophyceae) with reference to the utility of DNA barcoding. Mol Phylogenet Evol 44: 634-648.
124. McDevit DC SG (2009) On the utility of DNA barcoding for species differentiation among brown macroalgae (Phaeophyceae) including a novel extraction protocol. Phycological Research; 57: 131–141.
125. McDevit DC SG (2010) A DNA barcode examination of the Laminariaceae (Phaeophyceae) in Canada reveals novel biogeographical and evolutionary insights. Phycologia Volume 49, 235–248.
126. Bowers HA, Tomas C, Tengs T, Kempton JW, Lewitus AJ, et al. (2006) Raphidophyceae [Chadefaud Ex Silva] Systematics and Rapid Identification: Sequence Analyses and Real-Time Pcr Assays. J Phycol 42: 1333-1348.
127. Maistro S, Broady PA, Andreoli C, Negrisolo E (2007) Molecular phylogeny and evolution of the order Tribonematales (Heterokonta, Xanthophyceae) based on analysis of plastidial genes rbcL and psaA. Mol Phylogenet Evol 43: 407-417.
128. Cavalier-Smith T, von der Heyden S (2007) Molecular phylogeny, scale evolution and taxonomy of centrohelid heliozoa. Mol Phylogenet Evol 44: 1186-1203.
129. Hoef-Emden K, Kupper FC, Andersen RA (2007) Meeting report: Sloan Foundation Workshop to resolve problems relating to the taxonomy of microorganisms and to culture collections arising from the barcoding initiatives; Portland ME, November 6-7, 2006. Protist 158: 135-137.
130. Liu H, Probert I, Uitz J, Claustre H, Aris-Brosou S, et al. (2009) Extreme diversity in noncalcifying haptophytes explains a major pigment paradox in open oceans. Proc Natl Acad Sci U S A 106: 12803-12808.
